# Psychedelics in Mental Health: Opportunities and Challenges

**DOI:** 10.1002/brb3.71574

**Published:** 2026-07-09

**Authors:** Lena K. L. Oestreich, Nathalie M. Rieser

**Affiliations:** ^1^ School of Psychology The University of Queensland Brisbane Queensland Australia; ^2^ Australian Institute for Bioengineering and Nanotechnology The University of Queensland Brisbane Queensland Australia; ^3^ Center for Psychedelic and Consciousness Research, Department of Psychiatry and Behavioral Sciences Johns Hopkins School of Medicine Baltimore Maryland USA; ^4^ Pharmaco‐Neuroimaging and Cognitive‐Emotional Processing, Department of Adult Psychiatry and Psychotherapy University Hospital of Psychiatry Zurich, University of Zurich Zurich Switzerland

**Keywords:** implementation, mental health, psychedelics, psychotherapy, subjective experience, substance‐assisted therapy

## Abstract

Psychedelic and substance‐assisted therapies are attracting renewed scientific, clinical, and public interest as potential interventions for mental health disorders, particularly treatment‐resistant depression, post‐traumatic stress disorder, and substance use disorders. However, as the field moves from early efficacy trials toward clinical implementation, major questions remain regarding therapeutic mechanisms, psychological support models, safety, ethics, accessibility, and the role of subjective experience. This editorial introduces the *Brain and Behavior* special issue “Psychedelics in Mental Health: Opportunities and Challenges,” which brings together empirical studies, reviews, clinical perspectives, a case report, and commentaries addressing these emerging priorities. Across the special issue, a central theme is that psychedelic therapy cannot be understood as a pharmacological intervention alone. Contributions highlight the importance of therapeutic relationships, preparation, integration, music, touch, external therapist involvement, peer support, cultural context, and patient expectations in shaping outcomes. Several manuscripts examine the phenomenology of psychedelic and ketamine experiences, emphasizing that subjective meaning‐making, expectancy, and altered states are not incidental features but potentially central aspects of therapeutic change and clinical risk. Other articles consider implementation challenges, including models of care for veterans, the perspectives of peer support workers, global mental health equity, and culturally responsive access. Mechanistic and translational work further broadens the field by examining neuroplasticity, rapid‐acting interventions for suicidality, and developmental perspectives on psychedelic use. Collectively, the articles in this special issue illustrate a field at an important inflection point. Psychedelic research requires greater conceptual precision, careful attention to safety and consent, and implementation frameworks that prioritize equity and cultural appropriateness. The promise of psychedelics in mental health lies not only in their potential for rapid symptom reduction, but also in their capacity to challenge and expand prevailing models of therapeutic change, integrating biological, psychological, relational, cultural, and systems‐level perspectives on healing.

## Graphical abstract

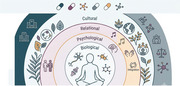

1

The renewed scientific and clinical interest in psychedelics has created one of the most dynamic areas of contemporary mental health research. After decades of regulatory, cultural, and scientific stagnation, substances such as psilocybin, lysergic acid diethylamide (LSD), ayahuasca, ketamine, 5‐MeO‐DMT, and 3,4‐methylenedioxymethamphetamine (MDMA) are once again being investigated for their potential to alleviate suffering across a range of psychiatric and psychological conditions. This resurgence has been driven by converging evidence suggesting that psychedelic and substance‐assisted interventions may produce rapid and clinically meaningful improvements in depression (Nutt et al. 2024; Haikazian et al. 2023; Metaxa and Clarke 2024), post‐traumatic stress disorder (PTSD) (Shahrour et al. 2024; Krediet et al. 2020; Brett and Bynum 2025), substance use disorders (Nutt et al. 2024; Rieser et al. 2022), anxiety (Muttoni et al. 2019), and existential distress (Schimmers et al. 2022; Marchi et al. 2024). Yet the field is also characterized by unresolved questions. What exactly constitutes “psychedelic‐assisted therapy”? Which components of the intervention are necessary, optional, or potentially harmful? How should subjective experience, expectancy, music, touch, preparation, integration, culture, and therapeutic relationships be understood and operationalized? How can emerging treatments be implemented safely, ethically, and equitably across diverse health systems?

This special issue, *Psychedelics in Mental Health: Opportunities and Challenges*, brings together empirical studies, reviews, commentaries, and clinical perspectives that collectively reflect a field in transition. The contributions move beyond the question of whether psychedelics may have therapeutic potential and instead ask how, for whom, under what conditions, and within which systems such interventions might responsibly be developed. A defining contribution of this special issue is its attention to the non‐pharmacological dimensions of psychedelic therapy. Although the public and scientific communities often focus on the compound, dose, or neurobiological mechanism, several articles underscore that the therapeutic process surrounding the drug may be equally central to outcome.

Murphy et al. (2026) argued directly against conceptualizing psychedelic‐assisted therapy as a standalone intervention. Drawing on the emerging clinical context in which patients may receive psychedelic‐assisted therapy while already engaged with psychiatrists, psychologists, psychotherapists, or other mental health professionals, a need for coordinated care between psychedelic therapy teams and external therapists was identified. Despite the centrality of psychotherapy and integration in most models of psychedelic care, there is no cohesive framework for collaboration between psychedelic therapy providers and a patient's existing treatment team. However, external therapists may play an important role across preparation, dosing, and integration, particularly for patients with treatment‐resistant conditions who have longstanding therapeutic relationships and complex clinical histories. The importance of relational and embodied care was further investigated by Ham et al. (2026) in a qualitative study of participant experiences of therapeutic touch during psilocybin‐assisted therapy. Touch is commonly included in psychedelic therapy manuals and clinical trial protocols, but it remains ethically sensitive, inconsistently defined, and under‐researched. In interviews with participants from a psilocybin‐assisted therapy trial, it was found that therapeutic touch was experienced heterogeneously. Although many participants valued its availability as a source of grounding, emotional connection, and affect regulation, others identified potential discomfort or distraction. Importantly, acceptability was closely linked to preparation, consent, trust, and the quality of the therapeutic relationship. These findings neither endorse touch uncritically nor reject it categorically. Instead, they point toward individualized, consent‐based, trauma‐informed protocols that treat touch as a clinical intervention requiring training, boundaries, and careful ethical governance. Music, another core feature of many psychedelic therapy protocols, was examined by Rowe et al. (2026) in a scoping review of quantitative evidence. The review identified only 19 articles comprising 330 participants, illustrating how limited the evidence base remains relative to the prominence of music in clinical practice. The available literature suggested that music may amplify emotion, engage brain networks involved in meaning attribution and imagery, and increase neural entropy, but direct causal evidence for an independent contribution of music to therapeutic response remains limited. A review by Grime and Drini (2026) highlighted that many contemporary debates around preparation, therapist presence, flexible dosing, therapeutic relationship, transference, and integration were already being explored during the first era of psychedelic research. Psycholytic therapy, in contrast to models oriented around a single peak or mystical experience, used LSD to facilitate ongoing psychoanalytically oriented therapy. The review identified procedural features associated with greater safety and effectiveness, including extensive preparation, therapist presence, flexible and intuitive therapeutic approaches, attention to transference, and creative integration. It also underscored that adverse outcomes were not absent historically and that risk was shaped by clinical practice, dose, supervision, and reporting standards. Together, these articles make a compelling case that psychedelic therapy is not simply “drug plus support.” Rather, it is a complex intervention in which pharmacological, psychological, relational, sensory, ethical, and contextual components interact dynamically.

A second major theme of this special issue is the need to take subjective experience seriously. Psychedelic research has often relied on symptom scales, diagnostic outcomes, or neurobiological measures, but several articles show that participant experience is not a secondary epiphenomenon. It may be a mechanism of change, a source of risk, a determinant of acceptability, and a methodological challenge. McGovern et al. (2025) analyzed online accounts of psychedelic experiences and identified a sequential structure: People describe their experiences in relation to what occurred before, during, and after psychedelic use. Prior to the experience, themes included subjective knowledge, perceptions of psychedelics, intention, mental preparation, and experiential aids. During the experience, themes included sensory and cognitive distortions, affective quality, mindset, and environmental support. Afterward, participants described impacts on behavior and outlook. This temporal structure mirrors the preparation‐dosing‐integration model used clinically, but it also expands it by showing how individuals themselves organize and interpret psychedelic experiences outside formal treatment settings. Poppe et al. (2026) extended this focus to clinical trial participation among individuals with treatment‐resistant depression. Through qualitative interviews with individuals considering participation in trials involving 5‐MeO‐DMT or psilocybin, motivations were shaped by hope, demoralization, prior psychedelic experience, and social factors. Expectations included anticipated symptom reduction, perceived mechanisms of change, the role of setting, and retrospective reinterpretation of expectations after participation. Particularly important was the observation that some participants viewed trial participation as a last resort following chronic illness and repeated treatment failures. This raises ethical and methodological challenges, whereby expectations may influence outcomes, but are also deeply human responses to suffering, hope, and limited treatment options. The role of subjective experience is also explored by Fritz et al. (2025) in a case report of a near‐death experience following emergency ketamine administration in the context of acute respiratory failure. The report carefully situates the patient's experience within a complex medical and pharmacological context, including hypoxia, hypercapnia, and high‐dose ketamine. The patient reported a vivid near‐death experience and later described positive psychological changes, including improved mood and empathy. Although causality cannot be disentangled, the case illustrates the clinical relevance of altered states that may occur outside psychiatric treatment settings and highlights the importance of recognizing, documenting, and sensitively discussing such experiences with patients.

The special issue also turns toward implementation: Who will deliver psychedelic‐assisted interventions, who will access them, and how will they be adapted across real‐world systems? Calnan et al. (2025) investigated the efficacy of naturalistic psychedelic retreats for military veterans, a population with high rates of PTSD, depression, reintegration difficulties, and unmet treatment needs. In a naturalistic retreat setting involving psilocybin or ayahuasca, veterans showed improvements across multiple outcomes, including depression, PTSD, sleep, well‐being, and post‐deployment reintegration. Notably, improvements were not limited to participants with PTSD diagnoses, suggesting that retreat‐based models may influence broader aspects of psychological and social functioning. The communal component of retreat settings may be particularly relevant for veterans, for whom social support, shared experience, and reintegration into civilian life are central to recovery. Lau et al. (2026) addressed implementation from the perspective of peer support workers. Their mixed‐methods study of Australian peer support workers found strong interest in psychedelic‐assisted therapy, while also identifying concerns around safety, stigma, psychoeducation, training, accessibility, and cost. Halm (2026) further argued that psychedelic‐ and substance‐assisted therapies risk remaining concentrated in high‐income countries unless research, training, policy, and implementation are deliberately expanded across diverse settings. The commentary also raised the ethical importance of cultural adaptation, especially given the long‐standing Indigenous and traditional uses of psychoactive plants such as ayahuasca, iboga, and psilocybin‐containing mushrooms. These implementation‐focused articles highlight that psychedelic‐assisted therapies are often resource intensive, involving specialist teams, preparation, dosing sessions, integration, medical oversight, and careful screening. Such intensity may be necessary for safety and efficacy, particularly in complex clinical populations. Yet it also creates barriers to scalability, affordability, and equity. If psychedelic treatments are to become part of mental health care, the field must address not only clinical efficacy but also workforce models, cost, cultural adaptation, regulation, community involvement, and access for underserved populations.

Although many articles in the issue focus on clinical process and implementation, others examine mechanistic and conceptual frontiers. Ekins et al. (2026) provided preclinical evidence that a single dose of the psychedelic neuroplastogen 25CN‐NBOH partially rescued intrinsic excitability deficits and restored synaptic drive in prefrontal cortex pyramidal neurons in a mouse model of prenatal alcohol exposure. This study extended psychedelic research beyond adult psychiatric symptom reduction and into developmental neurobiology and circuit repair. It reflects a growing interest in psychedelic‐inspired neuroplastogens, including compounds that may preserve plasticity‐promoting effects while reducing or eliminating hallucinogenic properties. Shah et al. (2026) conducted a systematic review and meta‐analysis comparing ketamine with midazolam for suicidality. Across randomized controlled trials, ketamine was associated with greater short‐term reductions in suicidal ideation and depressive symptoms than midazolam, although adverse effects, such as nausea, derealization, dizziness, and emotional disturbance, were more frequent. This review emphasized the potential role of rapid‐acting interventions in psychiatric emergencies, while also underscoring the need to clarify durability, safety, optimal dosing, and longer term treatment pathways. Payne et al. (2025) offered a broader developmental perspective in their integrative review of psychedelic use and positive adult development in emerging adulthood. It was proposed that classic psychedelics may interact with developmental trajectories related to identity, epistemic flexibility, meaning, relationships, ethics, and self‐insight. Importantly, the review acknowledged that emerging adulthood is also a period of heightened vulnerability for mental illness, substance‐related harms, psychosis‐spectrum disorders, and destabilizing experiences. Together, these articles demonstrate that the scientific horizon of psychedelic research is expanding. Psychedelics are being investigated not only as treatments for diagnosed disorders, but also as probes of neuroplasticity, consciousness, suicidality, development, meaning, and social functioning. This breadth is a strength, but it also demands conceptual clarity. The field must avoid collapsing distinct substances, mechanisms, populations, and intervention models into a single undifferentiated category of “psychedelics.”

Several cross‐cutting priorities emerge from this special issue. First, the field needs greater precision in defining interventions. “Psychedelic‐assisted therapy” or “substance‐assisted therapy” can refer to very different models: brief supportive therapy, structured manualized psychotherapy, psychodynamic psycholytic work, retreat‐based group processes, emergency ketamine administration, peer‐supported integration, or pharmacological neuroplasticity interventions. Without clearer description, reporting, discussion, and measurement of therapeutic components, it will remain difficult to determine that elements contribute to efficacy, safety, acceptability, and durability (Schettino et al. 2026; Richard and Levine 2025). Second, subjective experience must be integrated into research design. Expectancy, set, setting, music, touch, preparation, and integration are not merely confounds (Estric et al. 2025). They are active therapeutic contributions, ethical considerations, and potential mechanisms. At the same time, they complicate blinding, placebo control, and causal inference (Butler et al. 2022). Future trials should systematically measure expectations, therapeutic alliance, contextual features, participant narratives, and integration processes alongside conventional symptom outcomes (Murphy et al. 2021). Third, treatment standards must be addressed early rather than after efficacy is established. Workforce models, training standards, consent procedures, therapist competencies, peer roles, cost‐effectiveness, and care coordination are not peripheral issues (Kittur et al. 2025). They will determine whether psychedelic‐assisted therapies can be delivered safely and equitably. The articles in this special issue highlight that implementation requires adaptation to communities, health systems, cultures, and patient needs. Fourth, safety must remain central (Johnson et al. 2008). Enthusiasm for psychedelics is understandable given the burden of mental illness and the limitations of current treatments. However, the history of psychedelic research cautions against premature popularization, under‐reporting of adverse events, risks, and harms, and overextension beyond the evidence. Safety includes medical screening and acute monitoring, but also boundary management, trauma‐informed care, expectation management, long‐term follow‐up, and pathways for patients who experience adverse or destabilizing effects. Finally, equity must be treated as a scientific and ethical priority (Barber and Dike 2023). If psychedelic therapies are costly, urban, privatized, and available only to those with substantial resources, they may exacerbate existing mental health inequities (Adams et al. 2024). Conversely, if developed responsibly, they may offer new options for populations with high unmet need, including people with treatment‐resistant depression, PTSD, substance use disorders, suicidality, displaced populations, veterans, and those poorly served by existing systems.

The articles in this special issue collectively capture a field at an important inflection point. Psychedelic and substance‐assisted therapies hold considerable promise, but their future will depend on whether researchers, clinicians, policymakers, communities, and people with lived experience can move beyond simplistic narratives of breakthrough or backlash. The next phase of the field requires methodological rigor, conceptual nuance, ethical vigilance, and implementation science. The most compelling message of this special issue is that psychedelics in mental health care cannot be reduced to molecules, mystical experiences, or media narratives. They are complex interventions embedded in relationships, cultures, and systems. Their potential lies not only in rapid symptom change, but also in opening new ways of thinking about mental health treatment: as biological and psychological, individual and communal, clinical and cultural, scientific and deeply human. If the field can hold this complexity, psychedelic research may contribute more than a new class of interventions. It may help reshape how mental health care understands healing itself.

## Author Contributions

Lena K. L. Oestreich drafted the manuscript and Nathalie M. Rieser revised it. All author approved the final version of the manuscript.

## Funding

Lena K. L. Oestreich was supported by a National Health and Medical Research Council (NHMRC) Investigator Grant (Grant 2007718).

## Conflicts of Interest

The authors declare no conflicts of interest.

## Data Availability

Data sharing is not applicable to this article as no datasets were generated or analyzed during the current study.
